# Markers of Bone Metabolism Are Affected by Renal Function and Growth Hormone Therapy in Children with Chronic Kidney Disease

**DOI:** 10.1371/journal.pone.0113482

**Published:** 2015-02-06

**Authors:** Anke Doyon, Dagmar-Christiane Fischer, Aysun Karabay Bayazit, Nur Canpolat, Ali Duzova, Betül Sözeri, Justine Bacchetta, Ayse Balat, Anja Büscher, Cengiz Candan, Nilgun Cakar, Osman Donmez, Jiri Dusek, Martina Heckel, Günter Klaus, Sevgi Mir, Gül Özcelik, Lale Sever, Rukshana Shroff, Enrico Vidal, Elke Wühl, Matthias Gondan, Anette Melk, Uwe Querfeld, Dieter Haffner, Franz Schaefer, for the 4C Study Consortium

**Affiliations:** 1 Division of Pediatric Nephrology, Center for Pediatrics and Adolescent Medicine, Heidelberg, Germany; 2 Department of Pediatrics, University Hospital Rostock, Rostock, Germany; 3 Division of Pediatric Nephrology, Cukurova University, School of Medicine, Adana, Turkey; 4 Division of Pediatric Nephrology, Istanbul University Cerrahpasa Faculty of Medicine, Istanbul, Turkey; 5 Division of Pediatric Nephrology, Dpt. of Pediatrics, Hacettepe University Faculty of Medicine, Ankara, Turkey; 6 Division of Pediatric Nephrology, Ege University Medical Faculty, Izmir, Turkey; 7 Centre de Référence des Maladies Rénales Rares, Hôpital Femme Mère Enfant, Hospices Civils de Lyon, Bron, France; 8 Department of Pediatric Nephrology, University of Gaziantep, Gaziantep, Turkey; 9 Division of Pediatric Nephrology, University Children’s Hospital, Essen, Germany; 10 Division of Pediatric Nephrology, Istanbul Medeniyet University, Göztepe Training and Research Hospital, Istanbul, Turkey; 11 Diskapi Children’s Hospital, Ankara, Turkey; 12 Division of Pediatric Nephrology, Uludag University Faculty of Medicine, Bursa, Turkey; 13 Pediatric Hospital, University Hospital Motol, Prague, Czech Republic; 14 University Children’s Hospital, Hamburg, Germany; 15 KfH Kidney Center for Children, Marburg, Germany; 16 Department of Pediatric Nephrology, Sisli Educational and Research Hospital, Istanbul, Turkey; 17 Division of Pediatric Nephrology, Great Ormond Street Hospital, London, United Kingdom; 18 Dialysis and Transplantation Department of Pediatrics, Padova, Italy; 19 Department of Psychology, University of Copenhagen, Copenhagen, Denmark; 20 Department of Pediatric Kidney, Liver and Metabolic Diseases, Hannover Medical School, Hannover, Germany; 21 Integrated Research and Treatment Center Transplantation (IFB-Tx), Hannover Medical School, Hannover, Germany; 22 Clinic of Pediatric Nephrology, Charite Children’s Hospital, Berlin, Germany; Faculdade de Medicina Dentária, Universidade do Porto, PORTUGAL

## Abstract

**Objectives:**

The extent and relevance of altered bone metabolism for statural growth in children with chronic kidney disease is controversial. We analyzed the impact of renal dysfunction and recombinant growth hormone therapy on a panel of serum markers of bone metabolism in a large pediatric chronic kidney disease cohort.

**Methods:**

Bone alkaline phosphatase (BAP), tartrate-resistant acid phosphatase 5b (TRAP5b), sclerostin and C-terminal FGF-23 (cFGF23) normalized for age and sex were analyzed in 556 children aged 6–18 years with an estimated glomerular filtration rate (eGFR) of 10–60 ml/min/1.73m^2^. 41 children receiving recombinant growth hormone therapy were compared to an untreated matched control group.

**Results:**

Standardized levels of BAP, TRAP5b and cFGF-23 were increased whereas sclerostin was reduced. BAP was correlated positively and cFGF-23 inversely with eGFR. Intact serum parathormone was an independent positive predictor of BAP and TRAP5b and negatively associated with sclerostin. BAP and TRAP5B were negatively affected by increased C-reactive protein levels. In children receiving recombinant growth hormone, BAP was higher and TRAP5b lower than in untreated controls. Sclerostin levels were in the normal range and higher than in untreated controls. Serum sclerostin and cFGF-23 independently predicted height standard deviation score, and BAP and TRAP5b the prospective change in height standard deviation score.

**Conclusion:**

Markers of bone metabolism indicate a high-bone turnover state in children with chronic kidney disease. Growth hormone induces an osteoanabolic pattern and normalizes osteocyte activity. The osteocyte markers cFGF23 and sclerostin are associated with standardized height, and the markers of bone turnover predict height velocity.

## Background

Mineral and bone disorder (MBD) is a common complication of chronic kidney disease (CKD) in children. CKD-MBD encompasses complex abnormalities of bone turnover and mineralization, serum minerals and regulatory hormones, [1–3], may impact on linear growth and, by promoting vascular calcification, is considered a major cause of early cardiovascular morbidity in CKD. Treatment with recombinant growth hormone (rhGH) constitutes an approved and effective treatment for growth retardation in CKD. The effect of rhGH on bone metabolism is of particular interest since CKD-MBD and growth retardation are often connected.

However, monitoring of CKD-MBD is challenging. Bone biopsy is considered the gold standard procedure but, due to its invasiveness, currently recommended only in exceptional clinical situations [2]. Whereas plain X rays lack sensitivity, bone density measurements are more informative but still involve repetitive exposure to radiation. Hence, serum parathormone (PTH), calcium and phosphorus constitute the mainstay of CKD-MBD monitoring in routine clinical practice.

In 2006 KDIGO first recommended a detailed evaluation of emerging new biomarkers that may reflect bone cell activity in patients with CKD [1]. For children, the particular need to investigate potential associations to linear growth was emphasized. Here, we investigated in a large pediatric CKD population a panel of serum proteins potentially reflecting the activity of different bone cell types. Bone alkaline phosphatase (**BAP**), an osteoblast enzyme, is a sensitive and specific marker of bone formation and remodeling. The KDIGO 2009 guidelines suggest measuring either intact serum parathormone (iPTH) or BAP, since markedly altered levels are informative of bone turnover. In children BAP levels peak during infancy and puberty, mirroring the physiological activation of bone formation during these periods of rapid longitudinal growth. Tartrate-resistant acid phosphatase 5b (**TRAP5b**) degrades bone matrix proteins and is considered a specific marker of late osteoclast differentiation [4]. The peptide **sclerostin** is a novel key regulator of bone turnover [5]. It is released mainly by osteocytes as a paracrine negative feedback signal regulating bone formation by inhibiting the differentiation of osteochondral precursor cells to osteoblasts [6]. Finally, fibroblast growth factor 23 (**FGF-23)** is a key endocrine player in the regulation of bone mineral metabolism. Synthesized and released by osteocytes and osteoblasts [7] and targeting the renal tubule, FGF-23 plays an important role in maintaining mineral and vitamin D homeostasis.

Whereas the mechanisms and pathways of bone metabolism and the biological functions of the biomarker proteins are well established, their precise association to CKD-related abnormalities of bone metabolism and their role in height and growth of children with CKD is still controversial. Age- and gender-related differences and usually small available sample sizes add a further level of complexity to the interpretation of circulating bone markers in pediatric studies. Recently, pediatric reference values have been established for BAP, TRAP5b, sclerostin and c-terminal FGF-23 (cFGF-23), allowing age- and gender independent assessment of bone turnover in chronically diseased children [8].

The 4C Study consortium is following the largest cohort of children with CKD assembled to date [9]. We utilized the 4C cohort to characterize the patterns of these novel serum bone markers in pediatric CKD and to describe their associations with endpoints of statural growth, i.e. height standard deviation score (SDS) and its change over time, in children with and without concomitant recombinant growth hormone (GH) treatment.

## Methods

### Ethics statement

This study has been conducted within the framework of the 4C study. The 4C study was approved by the Institutional Review Boards (IRB) and Ethics Committees (EC) of all participating study sites. Informed written consent for participation in the 4C study and analysis of data and biosamples was obtained from parents/guardians on behalf of minors/children who also gave their consent to participate in the study. The study has been conducted according to the ethical principles of the Helsinki Declaration of 1964. The following Ethics committees (EC) and Institutional Review Boards (IRB) approved the 4C Study: Austria: EC of the University of Vienna; EC of the University of Innsbruck. Czech Republic: EC of the University of Prague. France: Comité de protection des personnes “Est IV”, Strasbourg. Germany: EC Charité—Universitätsmedizin Berlin; EC of the Medical Faculty of the University of Cologne; Ethikkommission der sächsischen Länderkammer; EC of Friedrich-Alexander-University Erlangen; EC of the University of Duisburg-Essen; EC of Albert-Ludwig University Freiburg; Ethikkommission der Ärztekammer Hamburg; EC and IRB of Hannover Medical School; EC and IRB of the University of Heidelberg; EC of the Medical Faculty of the University of Jena; EC of the Medical Faculty of the University of Leipzig; EC of Philipps University Marburg; Ethikkommission der Ärztekammer Westfalen-Lippe und der Medizinischen Fakultät der Westfälischen Wilhelms Universität Münster; EC of the Medical Faculty of the University of Rostock. Italy: Comitato Etico Indipendente dell’Azienda Ospedaliero-Universitaria di Bologna, Policlinico S.Orsola-Malpighi; Comitato di Etica dell’IRCCS Istituto Giannina Gaslini di Genova; Comitato Etico dell’IRCCS Ospedale Maggiore Policlinico, Mangiagalli e Regina Elena di Milano; Ethical Committee for clinical practice of the General Hospital-University of Padova; Comitato Etico per la Sperimentazione Clinica dell’IRCCS Ospedale Pediatrico Bambino Gesu’ di Roma. Lithuania: EC and IRB of Vilnius University. Poland: EC at Children’s Memorial Health Institute, Warsaw. Portugal: EC of the University of Porto. Serbia: EC of the University of Belgrade. Switzerland: EC of the Canton Bern; EC of the Canton Zurich. Turkey: EC of the University of Cukurova, Adana; Non-interventional Clinical Researches Ethics Boards of Hacettepe University, Ankara; EC of the University of Cerrahpasa, Istanbul; EC and IRB of the University of Ege, Izmir. United Kingdom: Great Ormond Street Hospital and UCL Institute of Child Health research EC.

### Study population

**Fig 1:**
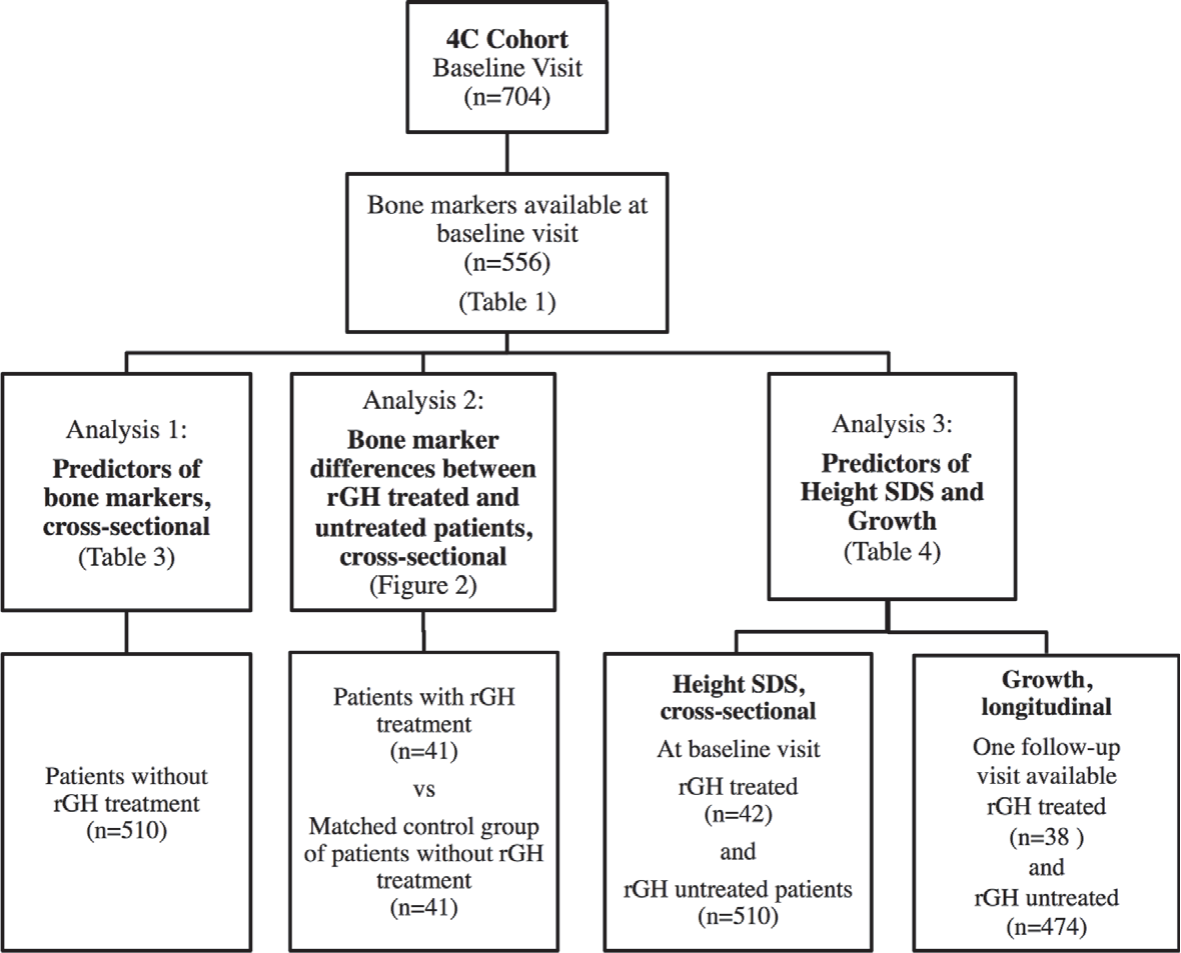
Patient inclusion criteria and subgroups for cross-sectional analysis of bone markers, influence of rhGH treatment and predictors of height and growth.

All patients were participants of the 4C Study (The Cardiovascular Comorbidity in Children with Chronic Kidney Disease Study, registered at ClinicalTrials.gov August 7, 2009, Identifier NCT01046448). The study is following children with CKD (eGFR 10–60 ml/min/1.73m^2^) aged 6–18 years. Clinical and biochemical examinations are performed every 6 months. Enrolment started in September 2009. All patients enrolled by December 2011 with available blood samples for bone marker analysis were included into this substudy of bone marker analysis. Bone markers were analyzed cross-sectionally. For the analysis of predictors of longitudinal growth, a follow-up clinical visit 6 (or alternatively 12) months after time of blood sampling was mandatory. Separate analyses were performed in order to 1) cross-sectionally analyze CKD-related predictors of bone markers, 2) compare bone markers of patients with rhGH treatment to a matched control group without rhGH treatment, and 3) analyze the influence of CKD-related factors and rhGH treatment on height and longitudinal growth. For patient selection and criteria for each analysis see Fig. 1.

The bone marker panel was measured in the baseline visit blood sample, concomitantly with a centralized biochemical analysis including serum creatinine, cystatin C, urea, C-reactive protein (CRP), bicarbonate, iPTH, calcium, phosphate and 25-hydroxy-vitamin D (25OHD). Estimated GFR (eGFR, ml/min/1.73m^2^) was calculated from creatinine, cystatine c, urea and height [10]. CKD stage was defined according to KDOQI [11]. Hypocalcemia was defined as a serum calcium level below 2.0 mmol/l; hyperphosphatemia was defined as serum phosphorus >1.87 mmol/L for patients aged 6 to 12 years and >1.45 mmol/L for those aged 12 years and older. A detailed patient and medication history was recorded. Physical activity was categorized into four levels depending on the hours of sports and other strenuous physical activity performed per week (0: none, 1: 1–2 hours/week, 2: 3–4 hours/week, 3: >4 hours/week). Patients were classified as rhGH treated or untreated when treatment modality was consistent over at least 3 months before bone marker analysis.

Renal diagnoses were categorized as CAKUT (congenital anomaly of the kidney and urinary tract), glomerulopathies (congenital/infantile/syndromal nephrotic syndrome, minimal-change nephropathy, focal segmental glomerulosclerosis, membranous nephropathy, mesangioproliferative glomerulonephritis, membranoproliferative glomerulonephritis, rapidly progressive glomerulonephritis, post-infectious glomerulonephritis), nephropathies due to systemic inflammatory disorders (Wegener’s granulomatosis, systemic lupus erythematosus Henoch-Schonlein purpura nephritis and IgA nephropathy), hemolytic uremic syndrome (HUS), metabolic disorders (cystinosis, oxalosis, nephrocalcinosis), other disorders (interstitial nephropathy, renovascular disease, post-ischemic chronic renal failure, other), or unknown.

### Laboratory analyses

ELISA kits were used for quantitative determination of cFGF-23 (Immutopics, San Clemente, CA, USA), sclerostin (Tecomedical, Sissach, Switzerland), BAP (Quidel, San Diego, CA, USA) and TRAP5b (Quidel). All assays were performed essentially as described by the manufacturers and all samples were assayed in duplicate. The inter-/intra-assay coefficients of variation for cFGF-23, sclerostin, BAP and TRAP5b were 5.1%/5.0%; 9.3%/5.9%; 5.5%/5.0%; and 4.2%/2.8%, respectively. If duplicate results differed by more than 5% (sclerostin: 7%), measurements were repeated on a second aliquot.

### Biostatistical approaches

The bone markers were normalized and standard deviation scores (SDS) were calculated for age and sex as proposed by Fischer et al., whose results on reference values were based on values obtained from 424 healthy children aged 0.1 and 21 years [8].

The influence of renal function and CKD stage on subject characteristics was tested by ANOVA for CKD stage, correlation analysis for eGFR and Kruskal-Wallis and chi square tests for categorical variables. The association of sex, age, BMI SDS, physical activity, metabolic status (serum calcium, bicarbonate, phosphorus, 25OHD) and renal function (eGFR, albuminuria) with standardized bone marker concentrations was evaluated by stepwise multivariate regression analysis in patients without rhGH treatment. For the assessment of the association of bone markers with longitudinal growth, the prospective change in height SDS was calculated over 12 months whenever available or annualized from the 6-month change if a complete 12-month follow-up was not available. The predictive value of serum bone markers on height and growth were tested by stepwise multivariate regression analysis correcting for age, sex, metabolic parameters and renal function in patients with and without rhGH treatment using rhGH treatment as confounding variable.

In all multivariate analyses, variables with a p value <0.15 were kept in the model during the selection procedure. Variables were tested for normality and log-transformed in case of violation of normality assumption.

To compare bone marker levels of patients with and without rhGH treatment with the best possible exclusion of confounding factors, the group of patients with rhGH treatment were compared to an untreated control group matched individually for age, sex, eGFR, CRP, and iPTH. Of the original cohort, the control group was selected out of 186 patients who were not treated with rhGH and were living in the same countries as children with rhGH treatment. Matching of rhGH treated and untreated patients was performed using SAS 9.2. Matches were available for 41 out of 42 rhGH treated patients. Post hoc testing for significant differences of matching variables and additional confounding factors between groups was performed.

Paired t-tests were applied to test for differences of bone marker levels between the matched groups. Statistical analyses were performed using SAS 9.2 and R. A p-value ≤ 0.05 was considered statistically significant.

## Results

### Subject characteristics

A cohort of 556 children and adolescents (365 males) participating in the 4C Study was studied. The patients were enrolled at 55 centers in Turkey (50%), Germany (15%), France (9%), the UK (6%), Italy (5%), Poland (4%), Austria (2.5%), Serbia (2.5%), Lithuania (2%), Switzerland (2%), Portugal (1%), and the Czech Republic (1%). Subject characteristics are given in Table 1. Underlying diseases comprised CAKUT in 74%, glomerulopathies in 6%, metabolic diseases in 3.5%, HUS in 2%, systemic vasculitis in 1%, others in 9.5% and unknown in 4% of the patients, respectively. The prevalence and severity of albuminuria (p<0.0003), hyperphosphatemia (p<.0001), hyperparathyroidism (p<0.0001), and metabolic acidosis (p<0.0009) increased with decreasing eGFR, whereas serum albumin, 25OHD, and CRP were not statistically significantly associated with renal function. Children with advanced CKD stages were shorter in stature (p<0.0001), but growth rates as expressed by the prospective change in height SDS did not differ significantly between CKD stages (p = 0.32).

**Table 1:** Subject characteristics.

	All		CKD Stage	
		3	4	5
N	556	219	296	41
Age (years)	12.2 ± 3.3	12.3 ± 3.2	12.2 ± 3.4	12.1 ± 3.1
% male	66	67	66	59
CKD duration (years)	6.0 ± 4.6	6.1 ± 4.7	6.3 ± 4.5	4.3 ± 4.1
BMI SDS	0.08 ± 1.28	0.02 ± 1.39	0.12 ± 1.24	0.15 ± 0.97
Height SDS	-1.38 ± 1.39	-1.05 ± 1.31	-1.58 ± 1.39	-1.64 ± 1.47
∆ height SDS (per year)	-0.04 ± 0.38	-0.02 ± 0.38	-0.03 ± 0.37	-0.13 ± 0.49
Midparental height (cm)	170.7 ± 9.4	171.3 ± 10.3	170.6 ± 9.2	168.7 ± 9.97
Physical activity level				
0	133 (23.9%)	43 (19.6%)	71 (24%)	19 (46.3%)
1	111 (20.0%)	39 (17.8%)	65 (22%)	7 (17.1%)
2	79 (14.2%)	37 (16.8%)	40 (13.5%)	2 (4.9%)
3	231 (41.6%)	99 (45.2%)	119 (40.2%)	13 (31.7%)
				
Serum bicarbonate (mM)	21.2 ± 3.8	21.6 ± 3.5	21.0 ± 3.8	20.5 ± 4.4
Serum calcium (mM)	2.21 ± 0.23	2.20 ± 0.21	2.22 ± 0.23	2.22 ± 0.27
Hypocalcemia	74 (13.3%)	31 (14.6%)	38 (12.8%)	5 (12.2%)
Serum phosphate (mM)	1.54 ± 0.37	1.47 ± 0.36	1.63 ± 0.35	1.67 ± 0.64
Hyperphosphatemia	183 (32.9%)	54 (24.7%)	102 (18.4%)	27 (65.9%)
Serum iPTH (pmol/l)	12.3 (7.1, 20.2)	9.2 (5.9, 15.2)	14.6 (8.7, 23.9)	17.8 (9.4, 36.3)
Serum 25OHD (μg/l)	11.0 (6.6, 18.1)	12.4 7.34, 17.6)	10.7 (6.4, 18.3)	9.2 (5.5, 20.0)
CRP (mg/l)	0.56 (0.22, 2.06)	0.55 (0.21, 2.01)	0.55 (0.3, 2.06)	0.98 (0.22, 3.16)
Albuminuria (mg/g creatinine)	331 (86, 1343)	192 (56, 749)	443 (112, 1669)	982 (359, 2330)
				
Medications				
rhGH	42 (7.6%)	11 (5.0%)	26 (8.8%)	5 (12.2%)
Vitamin D supplement	74 (13.3%)	21 (9.6%)	42 (14.2%)	11 (26.8%)
Active vitamin D analogue	281 (50.5%)	89 (40.8%)	165 (55.9%)	25 (61.0%)
Phosphate binders	254 (45.7%)	82 (37.6%)	142 (48.1%)	29 (70.7%)
				

Data are given as mean ± SD, median (interquartile range) or n (%) as appropriate.

At the time of bone marker assessment, 42 children (7.4%) were receiving rhGH treatment, excluding 4 patients who either started or stopped rhGH therapy within 3 months prior to analysis. 38 patients were consistently treated with rhGH during the follow-up period of 6–12 months. rhGH treated patients originated from 7 of the 12 participating countries, had a longer preceding duration of CKD (8.1±4.3 vs 5.9±4.5 years, p<0.003), were taller (-0.89±0.87 vs. -1.4±1.4 SDS, p<0.0002) and showed marginally better current growth rates (0.07±0.38 vs. -0.04±0.38, p = 0.08) compared to all non-rhGH treated patients.

### Effectors of serum bone marker concentrations

**Fig 2:**
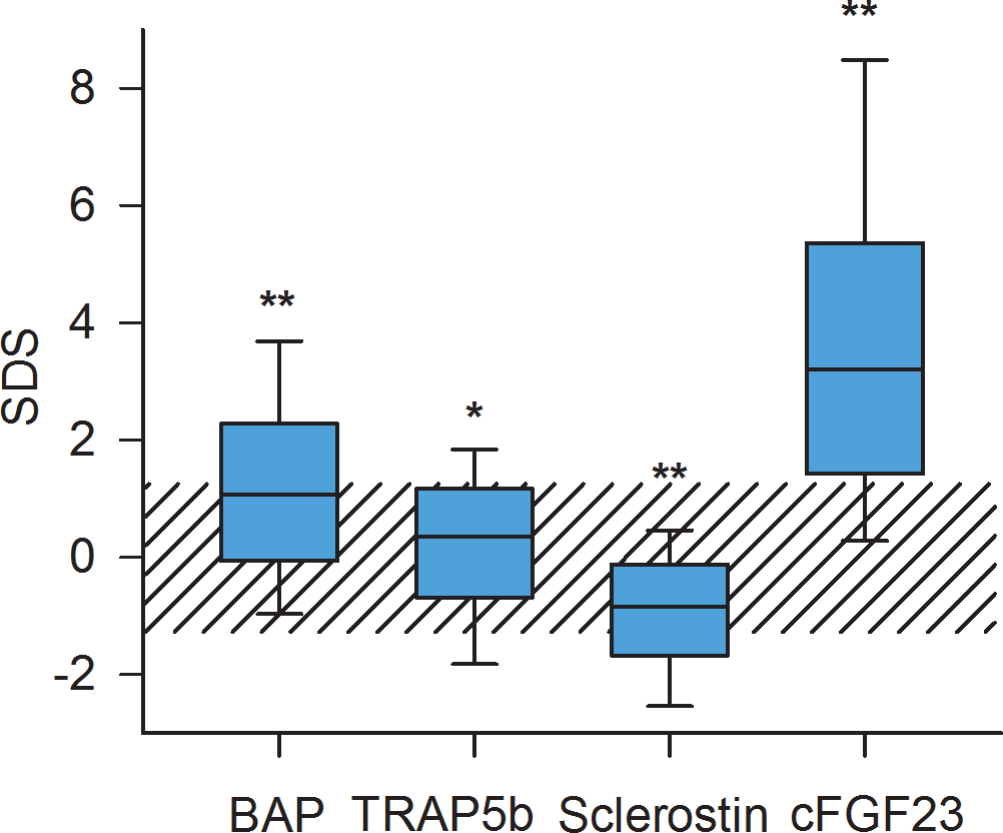
Distribution of serum bone marker concentrations in 510 pediatric CKD patients. Data are expressed as standard deviation scores (SDS). The shaded area depicts the normal range (5^th^ to 95^th^ percentile of biomarker concentrations in healthy children)[8]. Asterisks indicate significant deviation from distribution in the reference population (*: p<0.05, **: p<0.0001 compared to healthy controls).

Compared to healthy controls, standardized BAP, TRAP5b and cFGF-23 were significantly increased and Sclerostin decreased in the CKD population (Fig. 2 and Table 2). In univariate analysis BAP SDS was positively correlated to TRAP5b SDS (r = 0.58,p<0.0001) and inversely to cFGF-23 SDS (r = -0.2,p<0.0001). Sclerostin showed no correlation with TRAP5b, BAP or cFGF-23 (data not shown).

**Table 2:** Distribution of serum bone markers by CKD stage.

	All	p		CKD Stage	
			3	4	5
BAP (U/L)	147 ± 84		146 ± 74	151 ± 92	127 ± 73
BAP SDS	1.25 ± 2.06	<0.0001	1.27 ± 1.75	1.28 ± 2.27	0.91 ± 2.14
TRAP5b (U/L)	13.1 ± 6.6		13.3 ± 6.1	13.0 ± 7.0	12.8 ± 6.6
TRAP5b SDS	0.13 ± 1.56	0.05	0.18 ± 1.35	0.10 ± 1.69	0.16 ± 1.7
Sclerostin (μg/L)	0.31 ± 0.12		0.30 ± 0.12	0.31 ± 0.12	0.33 ± 0.16
Sclerostin SDS	-0.99 ± 1.17	<0.0001	-1.05 ± 1.16	-0.94 ± 1.12	-1.0 ± 1.6
cCFGF-23 (kRU/L)	183 (112, 321)		128 (89, 221)^A^	226 (136, 355)^B^	654 (321, 1224)^B^
cCFGF-23 SDS	3.21 (1.43, 5.46)	<0.0001	1.76 (0.67, 3.81)^A^	3.85 (1.95, 6.12)^B^	7.85 (4.94, 14.3)^C^

Data are given as mean ± SD or median (interquartile range) P values indicate difference of age- and sex-adjusted SDS from reference population. Different superscript letters indicate significant differences (p<0.05) between CKD stages (according to ANOVA using Student-Newman-Keuls grouping).

A, B & C: Student-Newman-Keuls Grouping—Means with the same letter are not significantly different.

In stepwise multivariate analysis, BAP SDS decreased and cFGF-23 SDS increased with declining eGFR, while TRAP5b SDS and Sclerostin showed no statistically significant association to renal function (Table 3). Albuminuria was associated with higher TRAP SDS and glomerulopathies as renal diagnosis were associated with lower BAP SDS. While inflammatory renal disease did not show any association to bone markers, BAP and TRAP5b appeared to reflect the impact of the current inflammatory status on bone metabolism as indicated by their independent associations with CRP levels. BAP and TRAP5b SDS were closely associated with iPTH (Table 3). Sclerostin SDS was predicted inversely by iPTH and positively by 25OHD levels. Patients with 25OHD deficiency (25OHD <16 μg/L) had significantly lower sclerostin and sclerostin SDS levels than patients higher 25OHD levels (sclerostin 0.30 vs 0.36 μg/l, p<0.0001 and -1.07 vs -0.61 SDS, p<.0001).

**Table 3:** Predictors of standardized bone marker concentrations; results of stepwise multiple linear regression analyses.

	BAP SDS			TRAP SDS			Sclerostin SDS			cFGF-23 SDS		
	ß ± SE	Part.R^2^	p	ß ±SE	Part.R^2^	p	ß ±SE	Part.R^2^	p	ß ±SE	Part.R^2^	p
Intercept	-2 ±0.75		0.008	-5.07 ±0.98		<0.001	-0.84 ± 0.45		0.06	-1.23 ± 1.98		0.53
Sex (m = 1, f = 2)	-0.35 ±0.18	0.01	0.02	0.32 ±0.14	0.005	0.005	-0.33 ±0.11	0.026	0.0004			
Age (y)	0.06 ±0.03	0.007	0.05	0.15 ±0.02	0.089	<0.0001				0.30 ±0.07	0.028	<0.0001
Phys Activity (0–3)	-0.17 ±0.07	0.01	0.02	-0.11 ±0.05	0.006	0.05						
eGFR (ml/min/1.73m^2^)	0.03 ±0.01	0.014	0.004	0.02 ±0.008	0.005	0.08				-0.21 ±0.02	0.13	<0.0001
Plasma iPTH (pM, log)	0.77 ±0.11	0.077	<0.0001	0.44 ±0.09	0.038	<0.0001	-0.26 ±0.06	0.045	<0.0001			
Serum Pi (mM)										1.77 ±0.68	0.015	0.004
Serum calcium (mmol/l)				0.55 ±0.33	0.005	0.1						
Serum 25OHD (µg/l)	0.23 ±0.12	0.07	0.05	0.16 ±0.09	0.006	0.09	0.22 ± 0.07	0.017	0.003			
Serum bicarbonate (mM)							0.03 ±0.01	0.006	0.08	0.22 ±0.06	0.025	0.0002
Serum CRP (mg/L, log)	-0.22 ±0.06	0.037	<0.0001	-0.20 ±0.04	0.054	<0.0001						
Albuminuria (g/g creatinine)				0.107 ±0.04	0.006	0.008						
Glomerulopathy	-0.65 ±0.37	0.076	0.04				-0.34 ± 0.23	0.004	0.13			
Model R^2^		0.173			0.22			0.106			0.2	

Serum cFGF-23 concentrations were strongly and independently affected by eGFR, serum phosphorus and bicarbonate levels. In contrast to the other bone markers, there was no association with iPTH or CRP.

### Serum bone markers and statural growth

Standardized cFGF-23 and sclerostin levels were independently positively associated with height SDS (Table 4). These relationships added independently to significant positive associations of age, eGFR and negative effects of metabolic acidosis on height SDS.

**Table 4:** Predictors of baseline and prospective change in standardized height in total cohort; results of stepwise multiple linear regression analyses.

	Height SDS		Change in height SDS per year	
	ß ± SE	p	ß ± SE	p
Age (y)	0.06 ± 0.02	0.0004	0.009 ± 0.001	0.09
Sex (male)	-0.52 ± 0.14	0.0002		
Height SDS	-		-0.03 ± 0.013	0.01
Midparental height (cm)	0.06 ± 0.008	<.0001		
BMI SDS	-0.09 ± 0.04	0.037		
Physical activity level (0–4)			0.05 ± 0.01	0.001
rhGH therapy			0.15 ± 0.07	0.04
BAP SDS			0.03 ± 0.01	0.002
TRAP SDS			0.03 ± 0.01	0.04
Sclerostin SDS	0.13 ± 0.05	0.005		
cFGF-23 SDS	0.04 ± 0.01	0.001		
eGFR (ml/min/1.73m^2^)	0.03 ± 0.007	<.0001	0.002 ±0.002	0.21
Serum bicarbonate (mM)	0.04 ± 0.015	0.01		
Serum Pi (mM)			0.14 ± 0.05	0.005
Serum 25OHD (μg/l, log)	0.006 ± 0.004	0.2	0.05 ± 0.02	0.04
Serum CRP (mg/L, log)	-0.07 ± 0.04	0.07		
Model R^2^	0.24		0.10	

The prospective change in height SDS during one year of observation was positively associated with higher BAP and TRAP5b SDS, lower baseline height SDS and rhGH therapy.

Interestingly, physical activity showed a positive association to prospective growth.

A case-control study was performed to obtain an unbiased analysis of the impact of rhGH on the bone marker pattern. For 41 rhGH treated patients an equal number of untreated control subjects matched by age, sex, country of residence, CKD duration, eGFR and serum phosphorus, iPTH and CRP was identified (see Table 5).

**Table 5:** Subject Characteristics for groups matched for GH treatment.

	GH untreated Controls	GH Treated Cases
n	41	41
		
Age (years)	11.9 ± 3.76	12.44 ± 3.24
Sex: male	35%	35%
CKD duration (years)	7.79 ± 3.73	8.16 ±4.37
		
Height SDS	-0.78 ± 0.91	-0.89 ± 0.88
∆ Height SDS	0.0 ± 0.39	0.06 ± 0.39
BMI SDS	0.1 ± 1.17	0.01 ± 1.27
		
eGFR (ml/min/1.73m^2^)	24.9 ±8.9	24.9 ± 8.95
Albuminuria (mg/g)	601 ± 899	1534 ± 5426
CRP (mg/dl)	0.57 ± 0.7	0.49 ± 0.55
Bicarbonate (mmol/l)	22.4 ± 3.0	21.7 ± 13.0
iPTH (pmol/l)	11.6 ± 6.4	12.0 ± 5.8
Calcium (mmol/l)	2.3 ± 0.2	2.29 ± 0.26
Phosphate (mmol/l)	1.60 ± 0.39	1.58 ± 0.27
25OH Vitamin D (μg/l)	16.9 ± 9.5	21.7 ± 13.0
1,25 OH Vitamin D (ng/l)	89.6 ± 73.0	88.6 ± 78.4
		
Diagnosis		
Glomerulopathies	5 (12%)	2 (5%)
CAKUT	30 (73%)	29 (71%)
Hemolytic Uremic Syndrome	2 (5%)	2 (5%)
Inflammatory	0 (0%)	1 (2%)
Metabolic	0 (0%)	1 (2%)
Others	4 (10%)	6 (15%)
		
Physical Activity Level		
		
0	14 (34%)	7 (17%)
1	9 (22%)	13 (32%)
2	7 (17%)	14 (34%)
3	11 (27%)	7 (17%)

**Fig 3:**
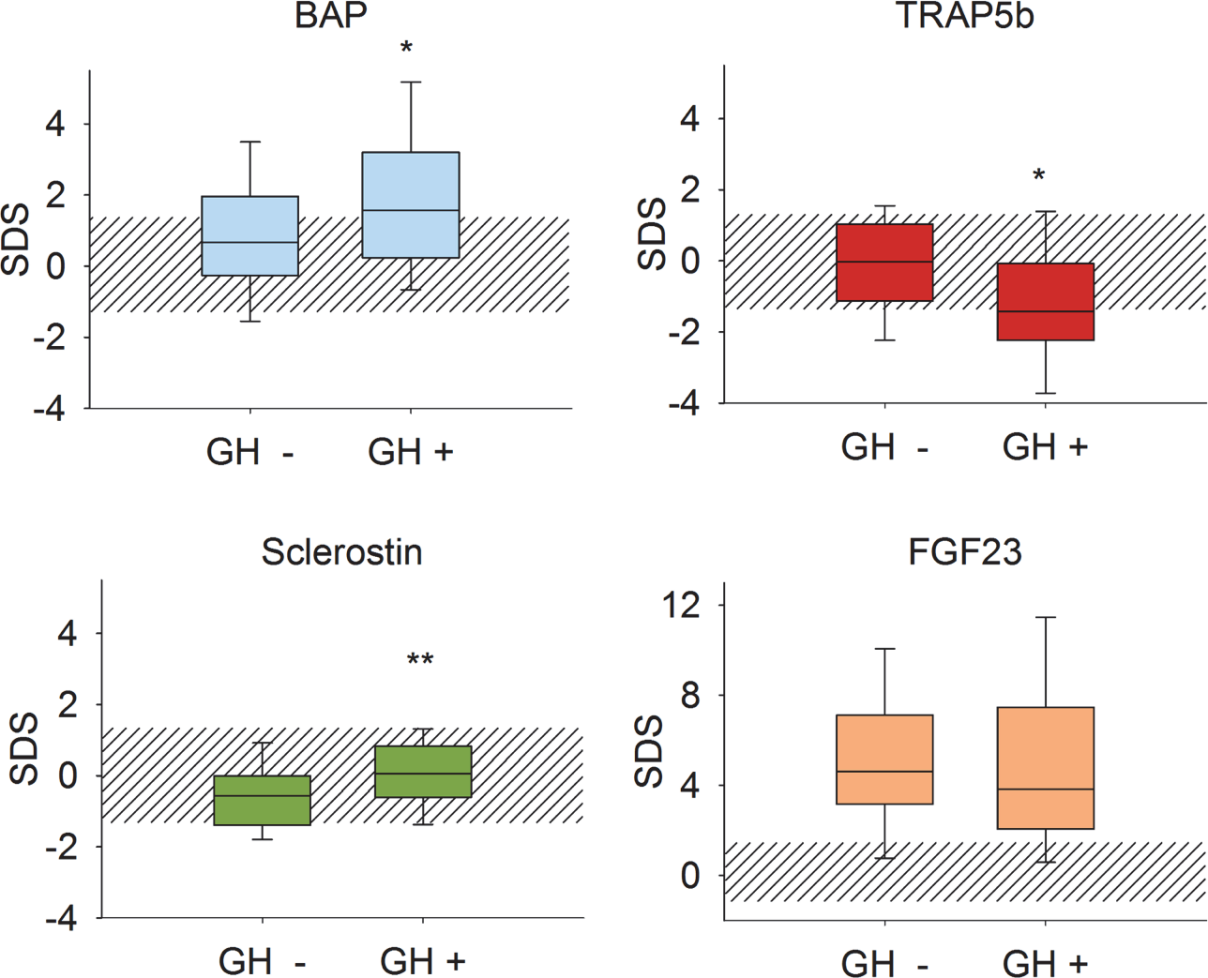
Distribution of serum bone marker concentrations in children with and without rhGH treatment (n = 41 per group). Data are expressed as standard deviation scores (SDS). The shaded area depicts the normal range (5^th^ to 95^th^ percentile of biomarker concentrations in healthy children)^8^. Asterisks indicate significant deviation from distribution in the reference population (*: p<0.05, **: p<0.01).

BAP SDS was significantly more increased in rhGH treated patients than in the untreated children (p<0.05) (Fig. 3, Table 6). Conversely, TRAP5b SDS was significantly lower in rhGH treated patients than in untreated controls (p<0.05). Sclerostin SDS in rhGH treated patients was not different from healthy controls but significantly higher than in the matched rhGH untreated patients (p<0.005). cFGF-23 did not differ by rhGH treatment status.

**Table 6:** Bone markers in groups matched for GH treatment.

	GH untreated group	GH treated group	p
BAP	144 ± 84	182 ± 87	0.03
BAP SDS	0.97 ± 1.93	1.93 ± 1.96	0.02
			
TRAP5b	11.9 ± 8.08	8.83 ± 4.82	0.03
TRAP5b SDS	-0.36 ± 1.96	-1.25 ± 1.75	0.05
			
Sclerostin	0.35 ± 0.16	0.46 ± 0.19	0.01
Sclerostin SDS	-0.63 ± 1.09	0.04 ± 0.96	0.005
			
cFGF23	407 ± 457	380 ± 417	n.s.
cFGF23 SDS	5.36 ± 4.07	4.72 ± 4.17	n.s.

## Discussion

This is the first comprehensive study of novel circulating markers of bone activity performed in a large, representative cohort of children with moderate to severe CKD. Within the pediatric age range, bone formation rate and bone turnover are essentially based on the developmental state of the skeleton. Relating our results to recently established age-specific reference values [8] allowed us to account for the physiological variability of bone markers during childhood. We demonstrated abnormal distributions of the osteoblast marker BAP, the osteoclast marker TRAP5b and the osteocyte markers cFGF-23 and sclerostin, compatible with a major alteration of bone turnover in this population. Furthermore, we observed significant changes of the bone marker profile in children undergoing rhGH treatment.

### BAP and TRAP

BAP levels are reflective of osteoanabolism and those of TRAP5b of bone resorption[12,13]. Both markers are correlated with serum intact PTH, but are superior to PTH in predicting bone histology and prospective changes in bone mass[13,14]. Previous studies of BAP and TRAP5B in children with CKD yielded heterogeneous results. In a small single-center study, BAP did not differ between children with CKD and healthy controls [15], possibly due to inclusion of a wide age range encompassing the physiological concentration peaks during infancy and puberty. For TRAP5b, elevated levels were found in children with CKD stage 5 but not in stage 3 and 4 [16]. Normalizing BAP and TRAP5B plasma levels for age and sex in this large pediatric cohort with CKD stage 3 to 5, we observed a clear increase of BAP and a slight but significant increase of TRAP5B levels. This finding is compatible with a preferential high bone turnover in this patient group. A small association of BAP SDS with eGFR emerged in multivariate but not univariate analysis, indicating a higher bone turnover rate in earlier CKD stages. However, the association was weak, suggesting a predominant role of factors other than renal function. When controlling for age, sex, eGFR and underlying renal disease the main predictors of increased BAP and TRAP5b levels were low CRP and high iPTH levels. The negative association of BAP and TRAP5b with CRP may indicate a link between inflammation and reduced bone turnover or adynamic bone disease independent of the underlying renal disease, whereas the independent positive association with iPTH supports a potential role of these bone markers for the assessment of high bone turnover.

### Sclerostin

In healthy children, sclerostin levels are higher in boys, decline during puberty and are positively correlated with the cortical porosity index [17]. In this pediatric CKD cohort, serum sclerostin levels were also higher in boys but did not change with age.

In adult CKD patients sclerostin levels increased progressively with advanced CKD [18]. However, in mice with progressive CKD sclerostin levels were found increased in early disease but decreased in advanced renal failure [19]. In keeping with that study we found overall reduced sclerostin levels in this cohort of patients with moderate to severe CKD. There is also experimental and clinical evidence of sclerostin suppression by PTH [19–21]. Accordingly, low sclerostin levels were predictive of high bone turnover in adults with CKD [22]. Our results confirm an inverse relationship of sclerostin and iPTH, adding to the notion that the osteoanabolic effects of iPTH may be partially related to the inhibition of sclerostin release.

Also, the bulk of sclerostin in healthy bone is released by osteocytes deep within the bone matrix whereas osteocytes near zones of bone remodeling produce little sclerostin [23]. Consistent with the observed high BAP and TRAP5b levels suggesting a high bone turnover state, the low plasma sclerostin concentrations in this cohort might indicate a relative preponderance of zones of bone remodeling over compartments with mature osteocytes and constant sclerostin production.

Little is known to date about the interaction of sclerostin with vitamin D metabolism. Increased 1,25-dihydroxy-vitamin D hydroxylase activity has recently been demonstrated in sclerostin knockout mice [24]. The independent positive association of 25OHD and sclerostin levels observed here might reflect the impact of vitamin D status on mineralized bone mass. Since the majority of children in this cohort were vitamin D insufficient or deficient, the decreased sclerostin levels might in part be explained by impaired vitamin D action.

### FGF-23

FGF-23 functions as a physiological endocrine regulator of serum phosphate and 1,25-dihydroxy vitamin D levels [25]. Here, we confirm for children the tight and independent regulation of circulating FGF-23 by eGFR and serum phosphate levels.

Plasma FGF-23 levels are age-dependent and increase early in the course of CKD prior to any abnormalities of serum phosphorus, calcium or PTH [8,26]. They correlate well with FGF-23 synthesis in the bone, as demonstrated in a study of 32 pediatric and young adult CKD patients [27]. FGF-23 is mainly expressed by osteocytes of the trabecular periphery. In dialyzed children circulating FGF-23 levels were found related to the process of osteoid maturation and mineralization time [28]. FGF-23 is highly expressed at sites of new bone formation [29] and appears to regulate osteoblast matrix mineralization in an age-dependent fashion [30,31]. In a study of predialysis children FGF-23, but not PTH or serum phosphorus, independently predicted bone formation [32]. All this evidence suggests a direct role of FGF-23 in bone formation and mineralization. While our study cannot provide insights into the bone morphology of children with CKD, it adds to the current state of knowledge the important observation that FGF-23 levels are associated with relative height in growing children. We also noted that patients with metabolic acidosis tend to exhibit lower FGF-23 at a given eGFR and serum phosphorus level. This association might indicate a novel mechanistic link between metabolic acdosis and mineral-bone disorder in CKD.

### rhGH promotes an osteoanabolic state in CKD

**Growth hormone** (GH) and its mediator IGF-1 drive longitudinal growth as part of their general anabolic action on tissues. CKD causes partial resistance to endogenous GH action, which can be overcome by administration of recombinant (r)GH at pharmacological doses. The 40 children receiving rhGH therapy in our cohort allowed to assess any effects of rhGH on bone metabolism. When compared to a matched control group of non-treated children, children receiving rhGH displayed significantly higher BAP and lower TRAP levels, consistent with an osteoanabolic action of rhGH.

This interpretation is supported by a randomized trial in dialyzed children investigating the effect of rhGH treatment on bone formation, where children receiving rhGH exhibited a higher bone formation rate than untreated children irrespective of bone histology at baseline [33]. Evidence for increased bone formation rate and bone mass from exposure to IGF1 also comes from conditions such as acromegaly and rhGH treated children receiving long-term corticosteroid therapy [34].

Sclerostin is a potent inhibitor of Wnt signaling, which plays an important role during bone formation. The finding that serum sclerostin levels were significantly higher with a distribution matching that of healthy children, suggests that rhGH might play a role in the regulation of bone formation via this pathway. We were unable to confirm the findings of a small study in GH deficient children showing an increase of cFGF-23 during rhGH supplementation therapy [35]; small changes in cFGF-23 might be overshadowed by the overall increase of cFGF-23 in CKD. Taken together, our findings support a major osteoanabolic effect of rhGH treatment in children with CKD.

### Bone markers predict height velocity

We confirmed several established causative factors as independently associated with small stature in CKD, such as low eGFR, metabolic acidosis, inflammation and vitamin D deficiency. Prospective catch-up growth was associated with rhGH treatment, a high physical activity level and higher serum phosphorus, the latter possibly reflecting good nutritional intake. When adjusting for these factors, Sclerostin and cFGF-23 SDS were independently associated with height SDS, and BAP and TRAP SDS were predictive of the prospective change in height. Similarly to this observation, a study in GH deficient patients showed an increase of BAP after treatment with rhGH, which was positively correlated with to improvement of height SDS and gain of bone density[36]. It is readily conceivable that Sclerostin and cFGF-23, representing cell activity in the mineralized bone compartment, be associated with relative body height, the final outcome of bone acquisition during statural growth, whereas BAP and TRAP as markers of bone remodeling rather show an association with short-term changes in growth velocity. Notably iPTH, the conventional biomarker of bone turnover, was not associated with either height or growth rate, confirming previous findings in dialyzed children [37].

In conclusion, in a large cohort of children with predialysis CKD we found important associations of serum bone biomarkers to patient characteristics, traditional markers of bone metabolism and linear growth. Our study benefited from a large sample size, the controlled observational study design and the application of age-specific reference values. However, a limitation results from the lack of a direct assessment of bone status by biopsy or skeletal imaging studies such as DEXA, pqCT or MRI. Also, it should be emphasized that the observed associations cumulatively explained only 10–20% of the overall variation of bone marker concentrations and 10–24% of the variance of growth indicators. Hence, while our study is informative regarding important biological regulators of bone metabolism and growth, other major sources of variation, including the impact of genetic disposition, remain to be explored. We hope that our findings will prompt further evaluation of these biomarkers including bone imaging techniques and bone histomorphometry to further validate their usefulness in the assessment and therapeutic monitoring of bone metabolism in children with CKD.
